# Clinical features and genotypes of Laing distal myopathy in a group of Chinese patients, with in-frame deletions of *MYH7* as common mutations

**DOI:** 10.1186/s13023-020-01626-y

**Published:** 2020-12-09

**Authors:** Meng Yu, Ying Zhu, Yuanyuan Lu, He Lv, Wei Zhang, Yun Yuan, Zhaoxia Wang

**Affiliations:** 1 Department of Neurology, Peking University First Hospital, No. 8 Xishiku Street, Beijing, 100034 China; 2 Department of Radiology, Peking University First Hospital, Beijing, China

**Keywords:** Laing distal myopathy, MYH7, Phenotype, Genotype, Pathology

## Abstract

**Background:**

Laing distal myopathy is a rare autosomal dominant inherited distal myopathy caused by mutations of the *MYH7* gene affecting mainly the rod region. We described the clinical features, muscle MRI and pathological changes as well as genetic mutations in a group of Chinese patients with Laing distal myopathy.

**Results:**

Six patients with the confirmed diagnoses of Laing distal myopathy were recruited. Ankle dorsiflexion and finger extension weakness, as well as neck flexion weakness were common in our patients. Myopathic as well as neurogenic lesions were suggested by electromyography in different patients. Respiratory abnormality of sleep apnea was detected in two of our patients stressing the necessity of close respiratory monitoring in this disease. Muscle MRIs showed similar features of concentric fatty infiltration of anterior thigh muscles together with early involvement of tibialis anterior and extensor hallucis longus. However, muscle pathological presentations were varied depending on the biopsied muscles and the severity of the disease. In-frame deletions of the *MYH7* gene made up 3/4 of mutations in our patients, suggesting that these are common mutations of Laing distal myopathy.

**Conclusions:**

Our study further expanded the phenotypes and genotypes of Laing distal myopathy. In-frame deletions of the *MYH7* gene are common causes of Laing distal myopathy.

## Background

Myosin is a family of highly conserved proteins acting as a molecular motor, which provides mechanical forces in a variety of cellular movements including muscle contraction [1]. Muscle myosin is myosin II consisting of two myosin heavy chain (MyHC) subunits and two pairs of light chain subunits [2]. There are several MyHC isoforms of striated muscles which are expressed in different muscle types. Among these, slow/β-cardiac MyHC is encoded by *MYH7*, and is mainly expressed in slow, type 1 muscle fibers as well as in heart ventricles [3].

Slow/β-cardiac MyHC can be divided into two parts: the N-terminal globular head region made by amino acids 1-847, and the C-terminal rod region made by amino acids 848-1935 [4]. A variety of both cardiac and skeletal muscle disorders can be caused by *MYH7* mutations. Mutations affecting the globular head region mainly cause cardiomyopathies, including familial hypertrophic/dilated cardiomyopathy, and left ventricular non-compaction (LVNC) cardiomyopathy, while mutations affecting the rod region mainly cause skeletal myopathies, including myosin storage myopathy (MSM), Laing distal myopathy (LDM) [5].

Laing distal myopathy is inherited as autosomal dominant with typical features of early-onset distal weakness mainly affecting ankle dorsiflexors and finger extensors, resulting in a “hanging big toe” sign. Neck flexion weakness is also very common in LDM. The disease course is usually very slow, with most patients remaining ambulant. Cardiac involvement is seldom seen in LDM, and serum creatine kinase (CK) levels are usually normal or only moderately elevated [6, 7]. Muscle MRI of LDM often shows the tibialis anterior as the earliest and most severely affected muscles [8]. However, no consistent conclusions of other muscles involvement on MRI are available. Muscle pathological changes in LDM are also variable and unspecific. Common pathological findings include fiber size variation, abnormally small type 1 fibers, type 1 or 2 fiber predominance, internal nuclei, minicores, and mitochondrial abnormalities [9].

Up to now, a number of both sporadic and familial LDM cases have been reported around the world, including several Chinese cases [10–12]. Here, we report three Chines LDM pedigrees and one sporadic Chinese LDM patient, and describe the clinical, radiographical, pathological and genetic features of these cases, to further expand both the genotypes and the phenotypes of LDM.

## Materials and methods

### Patients

We retrospectively reviewed patients with muscular disorders from Peking University First Hospital, and recruited patients suspected of Laing distal myopathy. All recorded clinical information was collected, including medical histories of clinical symptoms, distributions of muscle weakness, cardiac and pulmonary symptoms or evaluations, serum CK levels, and electromyography findings et al. Muscle strengths were evaluated with the Medical Research Council (MRC) scale. This study was approved by the Human Research Ethics Committee of Peking University First Hospital, and all participants provided written informed consent.

### Muscle MRI

Muscle MRIs using 1.5-T or 3.0-T magnetic resonance scanners (GE) of the thighs and/ or the calves were performed in five patients. Axial scanning in conventional T1-weighted and short T1 inversion recovery (STIR) sequences were carried out and additional images were acquired in the coronal plane when necessary. The detailed protocol for the scanning was according to our previous study [13]. The MRI findings were interpreted by an experienced radiologist and a neurologist independently and any disagreements were resolved by consensus.

### Muscle biopsy

Four patients received muscle biopsies. Muscle specimens were obtained from biceps in three patients, and from tibialis anterior in one patient. The muscle specimens were frozen in isopentane, and cooled in liquid nitrogen. Serial frozen sections were stained by routine histological and histochemical methods and by standard immunohistochemical techniques for dystrophin (N-terminus, C-terminus, and rod domain, Novocastra), sarcoglycan complex, (α-, β-, and γ-, Novocastra), dysferlin (Chemicon), and desmin (Novocastra).

### Genetic tests

Genomic DNA was extracted from peripheral blood samples of all enrolled patients and their available family members using standard procedures. Variants were detected by next-generation sequencing using the same protocol as previously described [14]. Sanger sequencing with specific primers was performed to confirm the selected variants. In addition, all the available family members of the patients were tested to confirm the segregation.

## Results

### Patients and clinical features

**Fig. 1:**
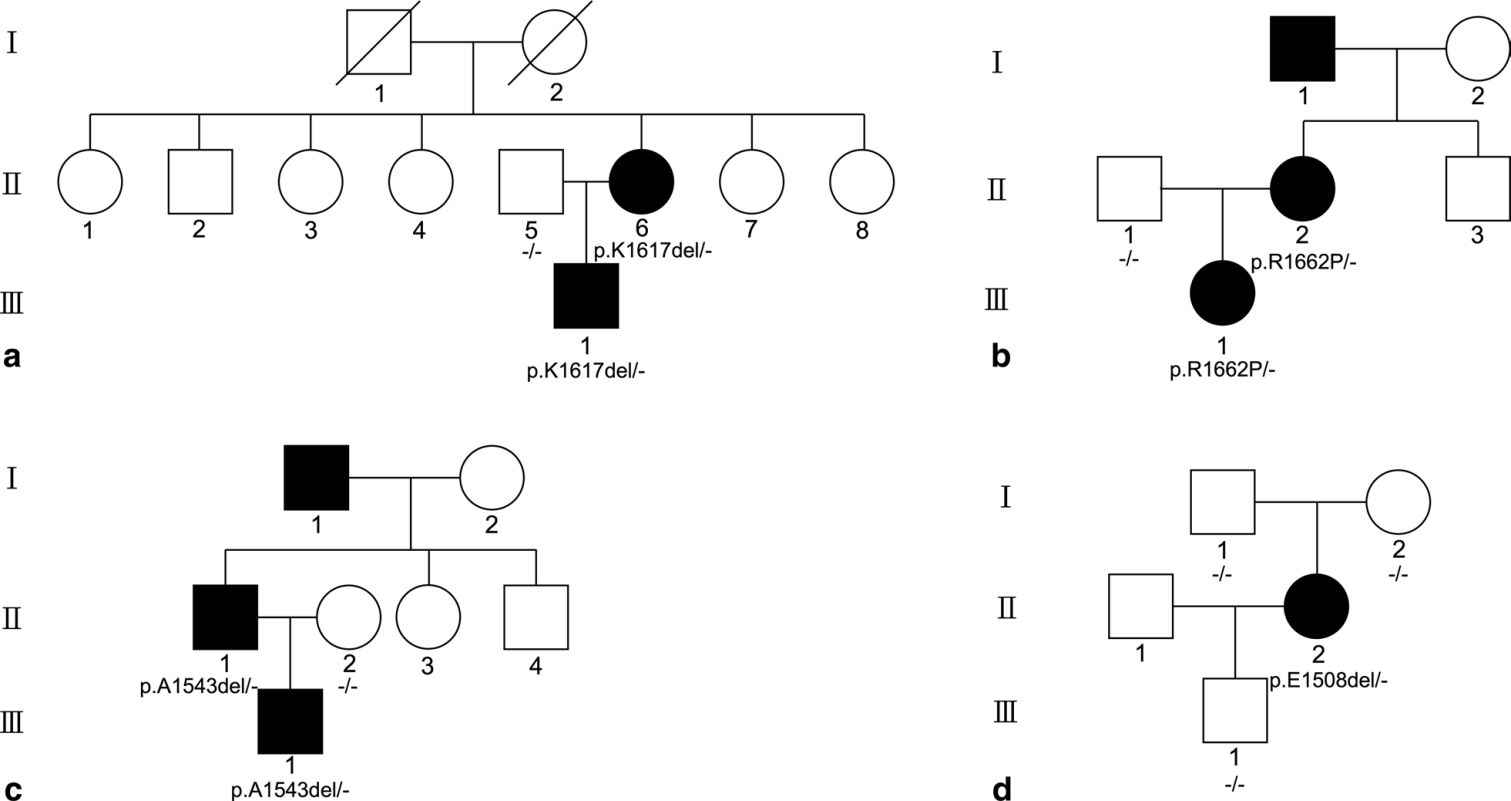
Enrolled familial and sporadic cases in the study

Five familial patients from three pedigrees and one sporadic patient with the diagnoses of Laing distal myopathy were recruited (Fig. 1). All six patients underwent routine cardiac and respiratory examinations, including electrocardiogram, Holter, echocardiography and pulmonary function test. Detailed clinical features were summarized in Table 1.

**Table 1:** Clinical features of the patients

No	Sex/age	Age of onset	Muscle strength*			Deep tendon reflexes	Skeletal deformities	Cardiac abnormalities	Respiratory abnormalities	CK U/L	EMG
			Neck flexion	Proximal limb	Distal limb						
A II-6	F/48	28	2	Upper: 4 Lower: 4-	Finger extension: 4- Ankle dorsiflexion: 0 Ankle plantarflexion: 3 +	Absent	Contracture of Achilles tendon, winging scapula	-	Sleep apnea	Normal	Myogenic
A III-1	M/13	8	4 +	Upper: 5 Lower: 4 +	Finger extension: 5 Ankle dorsiflexion: 4 Ankle plantarflexion: 5	Brisk	Contracture of Achilles tendon	-	-	Normal	N.A
B II-2	F/45	30	4	Upper: 5 Lower: 5	Finger extension: 4 Ankle dorsiflexion: 4 Ankle plantarflexion: 5	Brisk	-	-	-	Normal	Myogenic
B III-1	F/20	3	4 +	Upper: 5 Lower: 5	Finger extension: 3 Ankle dorsiflexion: 4- Ankle plantarflexion: 5	Brisk	Scoliosis, pes cavus	-	-	917	Neurogenic
C III-1	M/20	7	4	Upper: 4 Lower: 5	Finger extension: 4 Ankle dorsiflexion: 4- Ankle plantarflexion: 5	Absent	Contracture of Achilles tendon, scoliosis, winging scapula, strephenopodia	-	Sleep apnea	Normal	Neurogenic
D II-2	F/29	2	5	Upper: 0 (left)/ 5 (right) Lower: 4 +	Finger extension: 0 Ankle dorsiflexion: 0 Ankle plantarflexion: 5	Brisk	-	-	-	Normal	Myogenic

^*^All muscles strengths were evaluated with MRC scales

Patient II-6 and III-1 were from a large three-generation family A. Patient II-6 presented with abnormal toe walking at the age of 20, and found difficulty in climbing stairs at 31, then gradually showed finger weakness. She developed weakness in neck flexion and winging scapula at the age of 48. Examination at the age of 48 revealed significant decrease of muscle strengths in neck flexion (2/5), finger extension (4−/5), ankle dorsiflexion (0/5) and ankle plantarflexion (3 + /5), and mild decrease of proximal limb strengths (4/5 on upper limbs and 4−/5 on lower limbs) with all deep tendon reflexes absent. Contractures of Achilles tendons and winging scapula were present. No cardiac symptoms or abnormal examinations were found but with symptoms of sleep apnea. Serum CK was normal and EMG showed features of myogenic lesions. Patient III-1 was her 13-year-old son who developed bilateral Achilles tendon contracture since 8 years old. Strength tests only showed slight decrease in neck flexion, ankle dorsiflexion and proximal lower limbs. The rest family members were not affected.

Patient II-2 and III-1 were from a small three-generation family B. Father of patient II-2 was found abnormal gait since middle age but had not been examined or diagnosed. Patient II-2 only presented with slight symptoms of hand and foot weakness which did not affect her daily life. Examination showed slight decrease of muscle strengths in neck flexion (4/5), finger extension (4/5) and ankle dorsiflexion (4/5). No orthopedic, cardiac or respiratory abnormalities were found, and serum CK was also normal. Her daughter, patient III-1, presented with bilateral finger extension weakness since childhood, which progressed very slowly as she came to the clinic at the age of 20. Examination showed moderate decrease of muscle strengths in finger extension (3/5), and slight decrease of muscle strengths in neck flexion (4 + /5) and ankle dorsiflexion (4−/5). Scoliosis and talipes cavus were also present. Neither cardiac nor respiratory abnormalities were found. Serum CK was moderately elevated. EMG suggested neurogenic lesions with spontaneous activities as well as large motor unit potentials in several muscles, but nerve conduction studies were normal.

Patient III-1 was also from a three-generation family C. His grandfather was said to be weak in both legs while standing but was able to walk and climb stairs. His father presented with strephenopodia and abnormal gait since young. But neither of them was examined by clinicians. The onset symptoms of patient III-1 were strephenopodia and abnormal gait at the age of 7, and then he received orthopedic surgery. Examination showed slight decrease of muscle strengths in neck flexion (4/5), proximal upper limbs (4/5), finger extension (4/5) and ankle dorsiflexion (4-/5) with all deep tendon reflexes absent. Contracture of Achilles tendon, scoliosis and winging scapula were also present. Sleep apnea was obvious since 18 years old, so he had to use non-invasive positive pressure ventilation during sleep. No cardiac abnormalities were found, and serum CK was normal. EMG showed neurogenic lesions with spontaneous activities and large motor unit potentials in most of the muscles tested, but with normal nerve conduction studies.

Patient II-2 was the only symptomatic one in her family D, who could not run as fast as peers during childhood. After tug-of-war during high school, she was unable to lift her left arm. Examination at the age of 29 showed normal strength of neck flexion, but 0/5 in her left upper arm with normal strength in her right proximal arm. Muscle strengths of both fingers extension and ankles dorsiflexion were 0/5. No orthopedic, cardiac or respiratory abnormalities were found, and serum CK was normal. EMG showed myogenic lesions.

### Muscle imaging

**Fig. 2:**
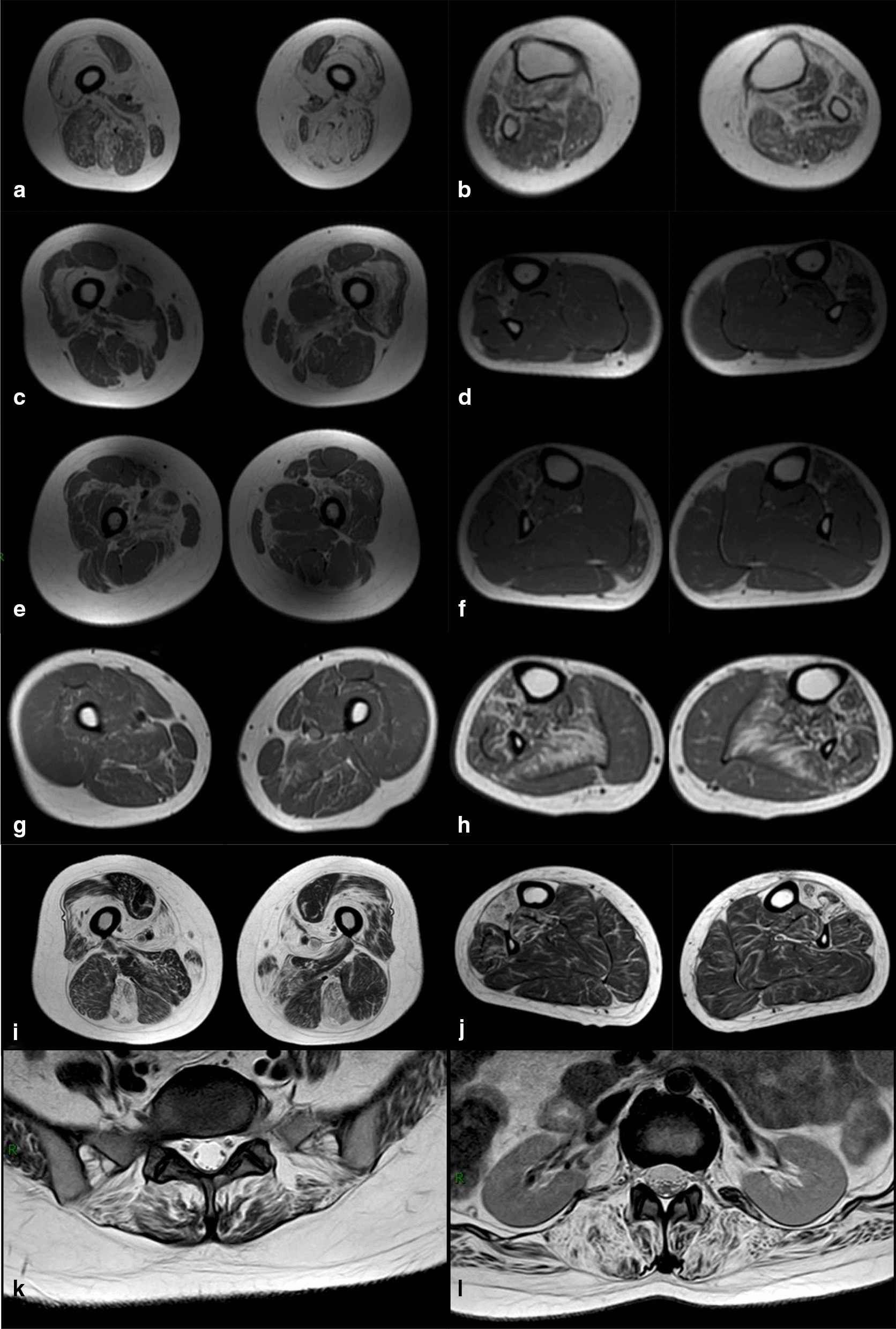
Muscle MRIs of the patients. **a**–**j** are lower muscle MRIs, each row belongs to one patient which are patient II-6 (family A), II-2 (family B), III-1 (family B), III-1 (family C) and II-2 (family D) respectively. Thigh muscle MRIs are on the left, and calf muscle MRIs are on the right. **k**–**l** are lumbar muscle MRIs of patient II-2 (family D). Detailed descriptions of the MRIs are present in the main text

Thigh and calf muscle MRIs were performed in all enrolled patients except patient III-1 (family A). The distributions of fatty infiltration were listed in Table 2 and shown in Fig. 2, which were not uniform among different patients. For thigh muscles, vastus intermedius and adductor magnus were severely affected in all the examined patients except patient III-1 (family C), in whom all thigh muscles were with diffusely mildly fatty infiltration. Other thigh muscles severely affected included semitendinosus in three patients, sartorius in two, semitendinosus in two, adductor longus in one and gracilis in one. Rectus femoris was relatively preserved in all examined patients, while other muscles were affected differently. For calf muscles, tibialis anterior and extensor hallucis longus were severely affected in all patients, while soleus was severely affected in two. Other calf muscles were mildly affected or relatively preserved. Patient II-2 (family D) also did lumbar MRI, which showed obvious fatty infiltration of paraspinal and other axial muscles (Fig. 2).

**Table 2:** Muscle MRI features of the patients

No	Age at MRI	Muscles with severe fatty infiltration	Muscles relatively preserved
A II-6	50	Thigh: gluteus maximus, vastus medialis, vastus lateralis, vastus intermedius, adductor magnus, sartorius, gracilis (left), posterior thigh muscles (left), semitendinosus (right) Calf: soleus (left), extensor digitorum longus, extensor hallucis longus, tibialis anterior	Thigh: rectus femoris, adductor longus Calf: soleus (right), peroneus longus, peroneus brevis, tibialis posterior
B II-2	45	Thigh: vastus intermedius, adductor magnus, semimembranosus Calf: tibialis anterior, extensor hallucis longus	Thigh: rectus femoris, adductor longus Calf: soleus, gastrocnemius, peroneus longus, peroneus brevis, tibialis posterior
B III-1	20	Thigh: vastus intermedius, adductor magnus (right) Calf: tibialis anterior, extensor hallucis longus	Thigh: rectus femoris, sartorius, gracilis, biceps femoris, semitendinosus, semimembranosus Calf: soleus, gastrocnemius, peroneus longus, peroneus brevis, tibialis posterior
C III-1	20	Thigh: diffuse mild fatty infiltration Calf: soleus, extensor digitorum longus, extensor hallucis longus, tibialis anterior, extensor hallucis longus	Calf: gastrocnemius, peroneus longus, peroneus brevis, tibialis posterior
D II-2	34	Thigh: vastus intermedius, adductor longus, semitendinosus, sartorius, gracilis Calf: tibialis anterior, extensor hallucis longus	Thigh: semimembranosus, biceps femoris, rectus femoris Calf: gastrocnemius, peroneus longus, peroneus brevis, tibialis posterior

### Muscle pathology

**Fig. 3:**
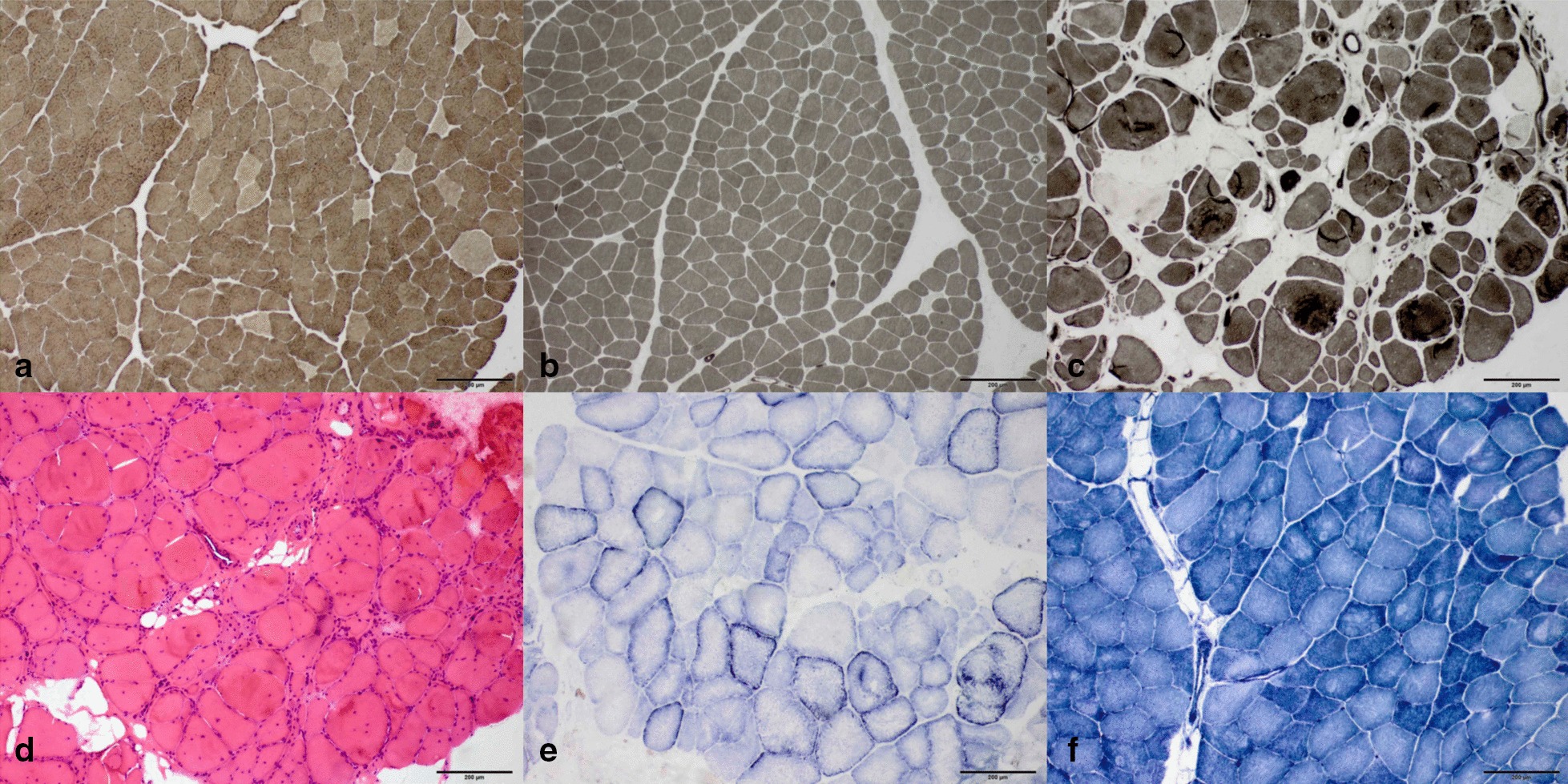
Muscle pathological changes of the patients. **a**–**c** are ATPase staining at pH 10.6, 10.7 and 10.6 of patient II-6 (family A), II-2 (family B) and III-1 (family C) respectively, showing type 2 fibers predominance. **c** also shows fiber type grouping. **d** is H&E staining of patient III-1 (family C) showing increased fiber size variation, necrotic and degenerated fibers. **e** is SDH staining of patient III-1 (family C) showing subsarcolemmal mitochondria accumulation. F is NADH-TR staining of patient II-2 (family D) showing multiple cores. All bar = 200 μm. NADH-TR, nicotinamide adenine dinucleotide tetrazolium reductase; SDH, succinate dehydrogenase

Muscle biopsies were done in patient II-6 (family A), II-2 (family B), II-2 (family D) on biceps and in patient III-1 (family C) on tibialis anterior. Pathological changes were summarized in Table 3. Increased fiber size variation was seen in all patients, while other common findings included type 2 fiber predominance in patient II-6 (family A), II-2 (family B) and III-1 (family C), nuclei internalization in patient II-6 (family A) and III-1 (family C). Specific findings included fiber type grouping and subsarcolemmal abnormal mitochondrial in patient III-1 (family C), as well as multi-cores and small type 1 fibers in patient II-2 (family D) (Fig. 3).

**Table 3:** Myopathological changes and *MYH7* mutations of the patients

No	Muscle biopsied	Age at biopsy	Pathological change	Nucleotide change	Amino acid change	Exon affected
A II-6	Biceps	48	Increased fiber size variation, nuclei internalization, type 2 fiber predominance	c.4850_4852delAGA	p.K1617del [5, 8]	34
A III-1	N.A			c.4850_4852delAGA	p.K1617del [5, 8]	34
B II-2	Biceps	45	Increased fiber size variation, type 2 fiber predominance	c.4985G > C	p.R1662P [15]	35
B III-1	N.A			c.4985G > C	p.R1662P [15]	35
C III-1	Tibialis anterior	20	Increased fiber size variation, nuclei internalization, type 2 fiber predominance, fiber type grouping, abnormal mitochondrial	c.4626_4628delAGC	p.A1543del	33
D II-2	Biceps	29	Multi-core, increased fiber size variation, small type 1 fibers	c.4522_4524delGAG	p.E1508del [16]	33

### Genetic findings

Mutations of *MYH7* were detected in all patients and not detected in non-affected family members (Table 3). Deletions of three nucleotides in exon 33 or 34 causing deletion of one amino acid were detected in family A, C and D. A missense mutation in exon 35 was detected in family B. Mutations in family A, B, and D had been reported as pathogenic in previous researches [5, 8, 15, 16]. The deletion mutation in family C was regarded as likely pathogenic according to ACMG guidelines.

## Discussion

*MYH7* mutations can cause two main groups of myopathies including myosin storage myopathy and Laing distal myopathy. In this study, we have described a series of patients with genetic confirmed Laing distal myopathy. Up to now, there have been more than 30 articles on Laing distal myopathy, including three reports on Chinese patients [10–12]. So far, this is the largest report on Laing distal myopathy in China. The onset ages are below 10 years old in young patients, but are 20–30 years old in middle-aged patients. This might be explained by the less awareness of weakness in patients or due to the relatively wide range of onset ages of this disease [9, 17]. The clinical features of this disease are characterized by slowly progressive muscle weakness of ankle dorsiflexion and finger extension [9]. The onset symptom of all six patients in our group is abnormal gait caused by ankle dorsiflexion weakness, suggesting its diagnostic value. Meanwhile, weakness of finger extension is also very common. Axial muscles involvement is another feature of this disease, which is also seen in our patients that five showed weakness of neck flexion and two showed scoliosis [18, 19]. Other orthopedic abnormalities including Achilles tendon contracture, winging scapula, and talipes cavus have also been reported in previous researches [5, 20].

None of our patients showed evidence of cardiac involvement, but two presented with obvious sleep apnea, and one of whom even needed ventilation assistance during sleep. This symptom has not been reported in previous researches, but should be paid more attention. Therefore, we suggest regular polysomnography test in patients with Laing distal myopathy to detect early sleep apnea. Deep tendon reflexes were absent in two patients, in accordance with neurogenic lesions on their EMGs, which suggests possible lower motor neuron or peripheral nerve involvement. High amplitude motor unit potentials and spontaneous activities on EMGs in Laing distal myopathy have also been pointed out in several reports, but without definite explanations [16, 17]. Muscle biopsy in patient III-1 (family C) showing fiber type grouping further supports the evidence of denervation.

Muscle MRI in our patients shows both similarities and distinctions. In the thigh level, there is a tendency of concentric distribution of fatty infiltration in anterior muscles with vastus intermedius more severe than other quadriceps. Adductor magnus is affected the most among thigh adductor muscles, but posterior thigh muscles are affected differently among patients. This distribution is similar to previous researches that vastus intermedius and lateralis were reported to be the most affected muscles followed by biceps femoris and semimembranosus, whereas the rectus femoris, adductor longus, semitendinosus, and gracilis were usually spared [8, 21]. In the calf level, tibialis anterior and extensor hallucis longus are the most severely affected, which is in accordance with clinical features of ankle dorsiflexion weakness and also the typical “hanging big toe” sign [9, 22]. The early involvement of tibialis anterior has also been reported in several researches, but our study further emphasizes extensor hallucis longus is also affected at the early stage [8]. Besides, one of our patients also presented with fatty infiltration of axial muscles, which was also seen in other studies [19]. Meanwhile, the asymmetrical involvement of both limbs is present in our patients, which is also seen in previous reports, and this could be another MRI feature of the disease [19].

Muscle pathological changes in our patients including type 2 fiber predominance, multi-cores and abnormal mitochondria, were relatively moderate and non-specific, which were in accordance with previous researches [8, 9, 17, 23]. Multi-cores were seen on muscle biopsy of patient II-2 (family D), and were also reported in some previous articles in patients with the same *MYH7* mutation p.E1508del [16, 24, 25]. This could be a specific pathological feature of this genotype. Other reported pathological features of the disease include central core, rimmed vacuoles et al. This suggests that muscle pathology could not be the definite diagnostic criteria of Laing distal myopathy but may only provide clues [26, 27]. Muscles selected for biopsy also affect the results that pathological changes of biceps were relatively slighter than tibialis in our study. Therefore, choosing more severely affected muscles for biopsy is also important, which can be assisted by MRI or other imaging techniques.

In four mutations of the *MYH7* gene detected in our patients, three are in-frame deletions. Referring to published articles on Laing distal myopathy, common mutation types also include missense mutations, and in-frame indels. The p.K1617del and p.E1508del have also been reported in a few studies [5, 6, 8, 9, 12, 16, 20–22].Besides these in-frame indels in our study, other reported mutations include p.E1687del, p.K1729del, p.E1669del, p.K1729dup, p.L1793del, p.K1784del [5, 9, 11, 17, 27–29]. Therefore, in-frame deletions or duplications are common types of mutations in Laing distal myopathy. One presumable mechanism is that the mutations may change the microsatellite allele size [6, 30].

## Conclusion

Our study further expands the clinical phenotypes and genotypes of Laing distal myopathy. In addition to muscle strengths and cardiac monitoring, we stress the importance of respiratory evaluation, especially polysomnography, in order to find early sleep apnea. Concentric fatty infiltration of anterior thigh muscles together with early involvement of tibialis anterior and extensor hallucis longus on MRI might be an imaging feature of the disease. Meanwhile, in-frame deletions of the *MYH7* gene are common causes of Laing distal myopathy.

## Data Availability

The datasets used and/or analyzed during this study are available from the corresponding author upon request.
